# Fibromatosis Colli: A Thorough Description of Its MRI Characteristics and a Review of the Literature

**DOI:** 10.5334/jbsr.3270

**Published:** 2024-05-07

**Authors:** Thomas Saliba, Marco Preziosi, Paolo Simoni, Alessandro De Leucio

**Affiliations:** 1 Queen Fabiola Children’s University Hospital, Av. Jean Joseph Crocq 15, 1020 Brussels, Belgium; 2 Queen Fabiola Children’s University Hospital, Av. Jean Joseph Crocq 15, 1020 Brussels, Belgium; 3 Queen Fabiola Children’s University Hospital, Av. Jean Joseph Crocq 15, 1020 Brussels, Belgium; 4 Queen Fabiola Children’s University Hospital, Av. Jean Joseph Crocq 15, 1020 Brussels, Belgium

**Keywords:** Fibromatosis colli, paediatric pseudotumor, pseudotumor, infant, MRI

## Abstract

**Introduction:**

Fibromatosis colli (FC) is a rare pseudotumor of the sternocleidomastoid muscle with an incidence of 0.4%, generally diagnosed using ultrasound between 2 and 4 weeks of age. This is an important entity considering the clinical concerns it causes due to its appearance as a cervical mass with torticollis. Few magnetic resonance imaging (MRI) descriptions of its appearance have been made, with the existing reported cases being sporadic. We aim to provide a thorough description of this paediatric entity.

**Materials and Methods:**

We conducted a retrospective study by searching our hospital’s database for previous cases of FC where an MRI had been performed. We found six cases of FC where an MRI had been performed. Of these cases, five out of six were contrast-enhanced. We examined the MRIs to be able to discern and describe the MRI characteristics of FC.

**Results:**

We found that FC presents a T1 signal isointense to the muscle, a T2 signal hyperintense to the muscle, a variable diffusion signal and a thick enhancing peripheral ring after contrast administration.

**Discussion:**

Our results match what has been reported in the literature to date regarding the MRI signal of FC, confirming previous reports. However, we provide new data regarding the characteristic appearance post-enhancement, which was previously unreported.

**Conclusion:**

The MRI characteristics of FC have rarely been described, with only a few isolated case reports in the medical literature. We review the current literature, describe the key MRI characteristics of the pathology, and provide the most thorough description to date.

## Introduction

Fibromatosis colli (FC) is a rare pseudotumor of the sternocleidomastoid muscle, with an incidence of 0.4% and a higher incidence in males (Alrashidi, 2022) [1]. It is currently classified as a benign myofibroblastic tumour according to the 2020 WHO classification [2]. The typical presentation is that of a child around 2 weeks old with a unilateral enlargement of the sternocleidomastoid muscle with subsequent torticollis [3–5]. The pathology seems to have a higher incidence in males and is the most common cause of perinatal neck masses [4]. This pathology has been reported to often be associated with birth trauma, with authors theorising that the fibrosis and subsequent shortening of the sternocleidomastoid muscle may occur after haemorrhage [1, 3]. FC has been reported to favour the right side in three quarters of cases, with its growth continuing for a few weeks after birth [3]. The head will typically be inclined towards the affected side, with possible rotation of the chin to the contralateral side [4, 5]. In 80% of cases, the pathology resolves within 2 years, generally being treated with conservative physiotherapy, but in some cases requiring surgical intervention [4].

## Materials and Methods

To conduct our retrospective study, we searched our department’s picture archiving and communication system (PACS) from the 1 January 2009 to the 1 April 2023. The search was conducted using the keywords “FC” and “fibromatosis colli.” In addition, we surveyed all the colleagues currently working in the department to find any additional cases that might have been missed in our search of the PACS. Our inclusion criteria were any magnetic resonance imaging (MRIs) in which the diagnosis of FC had been initially made, or subsequently confirmed, by ultrasound with a corresponding clinical history. Five of the exams were performed to investigate neck masses, the sixth exam was performed in the context of delayed development and hypertonia. Five of the MRI exams were performed using a Siemens Aera 1.5T MRI machine, and one exam was performed using a Philips Intera 1.5T MRI machine. One contrast-enhanced exam was performed using gadobenate dimeglumine (MultiHance®) contrast medium; the remaining four were performed using gadoterate meglumine (Dotarem®) contrast medium. The volume of contrast medium used was 0.2 mL/kg. The contrast medium was injected at a rate of 1 to 1.5 mL/s, and the acquisition was done in the venous phase for four exams, with a dynamic and delayed acquisition for the fifth exam. The exams were performed without sedation, but the children were fed beforehand in order to calm them.

## Results

The systematic retrospective search of our database yielded six cases upon which we based our retrospective study. Of these patients, five had previously undergone an ultrasound examination prior to the MRI, with one patient undergoing ultrasound follow-ups after the MRI. We summarised the patient’s ultrasound findings in Table 1.

**Table 1:** The ultrasound characteristics of the masses.

PATIENT NUMBER	SEX	AGE (MONTHS)	SIDE	LATERO-LATERAL LENGTH (MM)	ANTERO-POSTERIOR LENGTH (MM)	CEPHALO-CAUDAL LENGTH (MM)	ULTRASOUND FINDINGS
1	M	1.9	Right	12	7	25	Heterogenous sternocleidomastoid mass, diagnosed as fibromatosis colli. No adenomegaly.
2	M	2.17	Left	16	18	34	Heterogenous sternocleidomastoid mass, diagnosed as fibromatosis colli. No adenomegaly.
3	F	1.67	Left	12	12	23	Heterogenous mass seemingly adjacent to the sternocleidomastoid muscle, fibromatosis colli suggested as the most likely diagnosis. No adenomegaly.
4	F	1.23	Left	17	14	31	Heterogenous sternocleidomastoid mass, diagnosed as fibromatosis colli. No adenomegaly.
5	M	1.07	Left	17	33	32	Cervical mass is suspicious for neuroblastoma diagnosed in an external institution.
6	M	8.33	Right	11	11	21	No ultrasound before the MRI. Heterogenous sternocleidomastoid mass without adenomegaly at follow-ultrasound.

**Figure 1:**
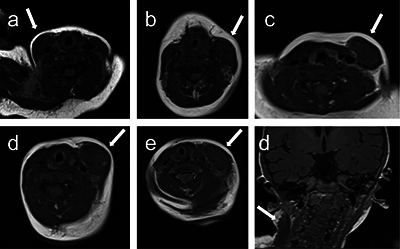
T1-weighted axial images from patients 1 to 5 (parts a–e) of the fibromatosis colli (arrows), isointense to the adjacent muscle. Part (d) is a T1-weighted coronal image of the fibromatosis colli (arrow) in patient 6.

**Figure 2:**
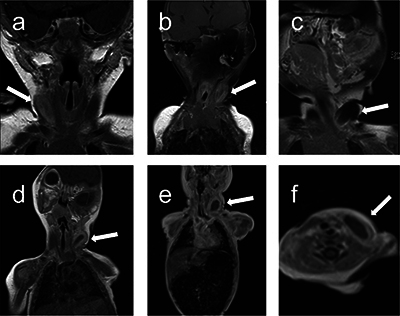
Contrast enhanced T1-weighted imaging, demonstrating the thick enhancing rim of the fibromatosis colli around a centre that remains isointense to the adjacent muscle (arrows). The coronal images (a) to (e) represent patients 1 to 5, with (f) being an axial reformatting of patient 5.

**Figure 3:**
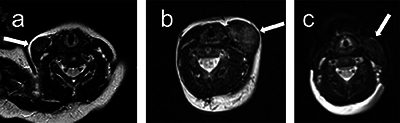
T2-weighted axial images from patients 1, 2 and 4 (labelled a–c) showing heterogeneous T2 hyperintensity of the fibromatosis colli (arrows).

**Figure 4:**
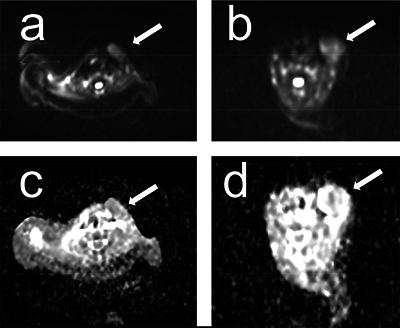
Diffusion weighted imaging (DWI b800) performed in patients 3 and 4 (parts a and b, respectively) showing hyperintensity of the fibromatosis colli (arrows) with accompanying low ADC values ranging from 1.25 to 1.4.

Because the MRIs were conducted over several years by different radiologists, using different MRI machines, and for different indications, the sequences used were not always identical, we have therefore defined the appearance of FC according to the most standard sequences (Table 2). No susceptibility weighted imaging (SWI) sequences were acquired. None of our patients presented with lymphadenopathy. Contrast-enhanced exams were performed for five of the six patients, as is standard in the workup of cervical masses in children [6]. The accompanying figures show the various images of these patients (Figures 1–4).

**Table 2:** The MRI characteristics using different weightings of the fibromatosis colli in each patient.

PATIENT	AGE (MONTHS)	CONTRAST ENHANCEMENT	DIFFUSION WEIGHTED IMAGING	T1 SIGNAL	T2 SIGNAL	FLUID SENSITIVE FAT-SATURATION SEQUENCE
**1**	1.9	Thick peripheral enhancement	Not performed	Isointense to muscle	Slightly hyperintense to muscle	STIR: Heterogenous hyperintensity compared to adjacent muscle
**2**	2.17	Thick peripheral enhancement	Not performed	Isointense to muscle	Heterogeneously hyperintense to muscle	T2 DIXON Fat-Sat: Heterogenous hyperintensity compared to adjacent muscle
**3**	1.67	Thick peripheral enhancement	Slightly hyperintense (ADC 1.25–1.4)	Isointense to muscle	Heterogeneously hyperintense to muscle	T2 DIXON fat-sat: Heterogenous hyperintensity compared to adjacent muscle
**4**	1.23	Thick peripheral enhancement	Slightly hyperintense (ADC 1.25–1.4)	Isointense to muscle	Heterogeneously hyperintense to muscle	T2 DIXON fat-sat: Heterogenous hyperintensity compared to adjacent muscle
**5**	1.07	Thick peripheral enhancement	No signal (artefact)	Isointense to muscle	Heterogeneously hyperintense to muscle	T2 DIXON fat-sat: Heterogenous hyperintensity compared to adjacent muscle with slight peripheral soft tissue oedema
**6**	8.33	Not performed	Not within field of view	Isointense to muscle	Isointense to muscle	Not performed

The first patient underwent an ultrasound for a sternocleidomastoid muscle mass, showing a heterogenous mass, which the radiologist diagnosed as FC. The clinicians requested an MRI to provide a better characterise the lesion as well as acquire measurements. The MRI confirmed the diagnosis of FC. A follow-up ultrasound was performed a month later, with a final ultrasound follow-up performed 11 months after the MRI, which was unchanged in comparison to the previous examinations. No further clinical follow-up occurred in our institution.

The second patient underwent an ultrasound for a suspected hematoma of the sternocleidomastoid muscle. The ultrasound was in favour of the diagnosis of FC, but an MRI was requested by the referring clinician due to the rarity of the pathology. The MRI confirmed the diagnosis of FC. The patient underwent physical therapy, with the torticollis resolving with no sequelae.

The third patient had a cervical mass for which an ultrasound was performed, revealing a heterogenous mass that did not seem to be part of the sternocleidomastoid muscle and was therefore atypical of a FC, though it was still suggested as the most likely diagnosis based on that exam. An MRI was performed at the request of the clinician, confirming the diagnosis of FC and the intramuscular location of the lesion. The patient underwent physical therapy with the torticollis resolving with no sequelae.

The fourth patient was referred for further imagery of a cervical mass, for which the ultrasound diagnosis of FC had already been made. However, the clinician wished to exclude a neuroblastoma or ganglioneuroma and requested an MRI of the cervical, thoracic and abdominal regions. The diagnosis of FC was confirmed by an MRI. The patient underwent physical therapy, with the torticollis resolving with no sequelae.

The fifth patient was a case referred for a cervical mass, based on an ultrasound from another institution, with a suspicion of a neuroblastoma. An MRI was therefore performed, excluding the neuroblastoma and having findings compatible with the diagnosis towards FC. The patient underwent physical therapy with the torticollis resolving with no sequelae.

The sixth case was a fortuitous discovery in a brain MRI of a child performed to exclude a cerebral anomaly. The child had a developmental delay, hypertonia and congenital torticollis. The presentation of the FC was slightly different in T2 compared to the other cases, possibly due to the patient being older. No contrast-enhanced T1-weighted imaging was performed in this patient. The patient underwent physical therapy, though the FC retained a heterogeneous aspect at a follow-up ultrasound 7 months after the initial MRI.

## Discussion

Whilst the physiopathology of FC is not yet fully elucidated, it has been theorised that it is linked to birth trauma, resulting in obstructed venous outflow from the muscle [1]. This, in turn, leads to necrosis and fibrosis of the muscle fibres, resulting in muscle strain [1]. The theory of venous outflow obstruction has some evidence in experimental studies and canine models [4]. Other theories link the pathology to the use of forceps, a breech position during birthing or primiparous births [4]. A competing explanation links the pathology to a sternocleidomastoid injury due to in-utero mispositioning of the foetuses’ head [1]. There have also been reports that the lesion appears in utero in 25% of cases, as well as reports of the condition in infants having been born by caesarean section [4].

FC can also be diagnosed using fine needle aspiration cytology, with some centres using this as the first line for diagnosis [7]. When this is performed, the typical presentation is that of spindled fibroblastic cells along with, sometimes necrotic, skeletal muscle cells [4, 7].

The pathology’s ultrasound characteristics the method mostly used to diagnose it, have been described in the literature as a diffuse enlargement within the sternocleidomastoid muscle appearing as either hyper or hypoechogenic compared to the surrounding muscle, moving synchronously with the muscle [3, 5]. One might find hyperechogenic foci representing post-haemorrhage calcifications [5]. If there is associated lymphadenopathy or the mass extends beyond the muscle, this should prompt further investigation due to it not being typical in FC cases [5].

When non-enhanced computed tomography (CT) scanning is used, although this is not the diagnostic tool of choice, FC presents as an isodense mass within the sternocleidomastoid muscle [8].

Due to the limited prevalence of this condition and the fact that the diagnosis is mainly obtained using ultrasound, the MRI characteristics have hitherto not been clearly defined, with only one previous article from 1998 in which only T1-weighted gradient recuperation imaging and T2-weighted characteristics were described [8]. A further article from 2022 also described the T2-weighted appearance of an isolated case, but once again, no contrast-enhanced sequences were performed [9]. One must note, however, that two other authors provided a T1-weighted imaging description three decades ago, in 1989 and 1994 [10, 11].

Upon examination of the six examples available to us, we found that FC appears on MRI as a thickening of the sternocleidomastoid muscle, often causing a marked inclination of the neck towards the affected side, giving a clue to its likely location. To facilitate its description, we have described its intensity compared to the adjacent sternocleidomastoid muscle. The FC is isointense to the muscle in T1-weighted imaging, giving the muscle a broader appearance. In T2-weighted imaging, the mass appears heterogeneously hyperintense to the surrounding muscle, with some strands of isointense fibres, with this appearance being similar in fluid-sensitive imaging with fat saturation. The FC mass appears slightly hyperintense in Diffusion weighted imaging, with corresponding Apparent diffusion coefficient values of 1.25 to 1.4. The most striking characteristic is its peripheral enhancement after contrast administration. The enhancement forms a thick rim around the isointense centre. In one of the cases we included in our retrospective analysis, perfusion imaging was performed, showing that the enhancement occurred relatively late.

Interestingly, some authors describe the mass as having a less hyperintense T2 signal compared to T1, which is the opposite of all six examples in our series [8]. However, another paper describes a T1- and T2-weighted appearance that matches our findings [12]. A final paper also described T2-weighted imaging in an isolated case, which matches our findings [9]. The only description of FC with contrast-enhanced MRI before our study was by Lowry et al., though they described an enhancing solid mass, which stands in opposition to the five cases that we described, where we found distinctive peripheral enhancement [4].

The treatment for this disease is generally physiotherapy, with the pathology tending to resolve within 2 years, though in some cases surgery is required [1, 4, 6, 13–16].

The differential diagnosis includes lipoma, haemangiomas, aggressive fibromatosis, teratomas and adenopathy, though with a proper clinical exam and accompanying imagery are generally being able to lead to the correct diagnosis [3, 5, 17]. Malignant causes of neck swelling include neuroblastoma, rhabdomyosarcoma and fibrosarcoma [4]. It should be noted, however, that none of these tend to present so early in childhood [4].

The differential diagnosis for masses with peripheral rim enhancement post-contrast is large, including abscesses, myositis, necrosis and sarcomas with necrotic centres [13]. The enhancement is dissimilar to that found in non-ossifying myositis, which typically presents with whole-lesion enhancement [14]. As such, it is important to consider the overall clinical context when making a definitive diagnosis based on an MRI.

Although the use of gadolinium contrast in paediatric patients is somewhat controversial due to the unclear long-term effects, it is generally considered safe, and as such, it is justified in cases where it may be beneficial to the diagnosis and, as such, is recommended for the workup of cervical masses [6, 15, 16].

Despite our work being the most thorough MRI description to date, we are also aware of the possible limitations of our results. The main limitation is that we were limited to six cases, which, despite the overall uniformity of the results, may result in the absence of possible variants that are not common enough to appear in our dataset. It should be noted, however, that although six cases may not appear to be a large sample, our retrospective study is the largest to date to look at the MRI characteristics of FC. This is due to the rarity of the pathology and the even greater rarity of using an MRI to define its characteristics. Another limitation is that the MRIs were performed by different radiologists and on different MRI machines over an extended period of time, leading to imperfect uniformity of the MRI study protocols used. However, despite the differences in methodology, we were able to demonstrate a consistency in the appearance of the FC between all six cases available. We do not, however, believe that MRI should be part of routine practice for the diagnosis of FC, with an ultrasound and clinical assessment being adequate in the vast majority of cases.

By means of our retrospective study, we found that FC is isointense to the muscle in T1-weighted imaging, hyperintense to the muscle in T2-weighted imaging, hyperintense in DWI and ADC maps, and has as a defining characteristic a thick peripherally enhancing rim. Ours is the first retrospective study to have such a large dataset of cases to draw data from, as previous MRI descriptions were limited to isolated sporadic case reports and therefore unable to draw broader conclusions. Although we believe that we have defined the MRI characteristics of the majority of cases of FC, we cannot exclude that there may be variants and therefore believe that more research may be required in order to further explore any outliers.

## Data Availability

Not applicable.
